# Youth at-risk for serious mental illness: methods of the PROCAN study

**DOI:** 10.1186/s12888-018-1801-0

**Published:** 2018-07-05

**Authors:** Jean Addington, Benjamin I. Goldstein, Jian Li Wang, Sidney H. Kennedy, Signe Bray, Catherine Lebel, Stefanie Hassel, Catherine Marshall, Glenda MacQueen

**Affiliations:** 1 Hotchkiss Brain Institute, Department of Psychiatry, University of Calgary, Mathison Centre, 3280 Hospital Dr NW, Calgary, AB, Calgary, AB T2N 4Z6 Canada; 2 Centre for Youth Bipolar Disorder, Sunnybrook Health Sciences Centre, Toronto, ON Canada; 3 Departments of Psychiatry and Pharmacology, Faculty of Medicine, University of Toronto, Toronto, Ontario Canada; 4 Work & Mental health Research Unit, Institute of Mental Health Research, University of Ottawa, Ottawa, Ontario Canada; 5 School of Epidemiology and Public Health, Faculty of Medicine, University of Ottawa, Ottawa, Ontario Canada; 6 Department of Psychiatry, University Health Network, Toronto, Ontario Canada; 7 Department of Psychiatry, St. Michael’s Hospital, Toronto, Ontario Canada; 8 Arthur Sommer Rotenberg Chair in Suicide and Depression Studies, St. Michael’s Hospital, Toronto, Ontario Canada; 9 Li Ka Shing Knowledge Institute, St. Michael’s Hospital, Toronto, Ontario Canada; 10 Krembil Research Institute, University Health Network, Toronto, Ontario Canada; 11 Department of Radiology, University of Calgary, Calgary, Alberta Canada; 12 Alberta Children’s Hospital Research Institute, Calgary, Alberta Canada; 13 Child & Adolescent Imaging Research (CAIR) Program, Calgary, Alberta Canada

**Keywords:** Depression, Bipolar disorder, Psychosis, Schizophrenia, Youth, Clinical high risk, Risk

## Abstract

**Background:**

Most mental disorders begin in adolescence; however, there are gaps in our understanding of youth mental health. Clinical and policy gaps arise from our current inability to predict, from amongst all youth who experience mild behavioural disturbances, who will go on to develop a mental illness, what that illness will be, and what can be done to change its course and prevent its worsening to a serious mental illness (SMI). There are also gaps in our understanding of how known risk factors set off neurobiological changes that may play a role in determining who will develop a SMI. Project goals are (i) to identify youth at different stages of risk of SMI so that intervention can begin as soon as possible and (ii) to understand the triggers of these mental illnesses.

**Method:**

This 2-site longitudinal study will recruit 240 youth, ages 12–25, who are at different stages of risk for developing a SMI. The sample includes (a) healthy individuals, (b) symptom-free individuals who have a first-degree relative with a SMI, (c) youth who are experiencing distress and may have mild symptoms of anxiety or depression, and (d) youth who are already demonstrating attenuated symptoms of SMI such as bipolar disorder or psychosis. We will assess, every 6 months for one year, a wide range of clinical and psychosocial factors to determine which factors can be used to predict key outcomes. We will also assess neuroimaging and peripheral markers. We will develop and validate a prediction algorithm that includes demographic, clinical and psychosocial predictors. We will also determine if adding biological markers to our algorithm improves prediction.

**Discussion:**

Outcomes from this study include an improved clinical staging model for SMI and prediction algorithms that can be used by health care providers as decision-support tools in their practices. Secondly, we may have a greater understanding of clinical, social and cognitive factors associated with the clinical stages of development of a SMI, as well as new insights from neuroimaging and later neurochemical biomarker studies regarding predisposition to SMI development and progression through the clinical stages of illness.

**Electronic supplementary material:**

The online version of this article (10.1186/s12888-018-1801-0) contains supplementary material, which is available to authorized users.

## Background

There has been a great deal of focus on risk for psychosis with many studies focussing on young people who are at clinical high risk of psychosis [1]. However, serious mental illness (SMI), in general, including bipolar disorder and recurrent major depression along with psychosis has drastic individual, family and societal consequences. It is important to address the risk for any SMI in youth because (i) most mental disorders begin in adolescence, with 75% of chronic and persistent SMI starting between the ages of 10 and 24 [2], (ii) mental disorders are the main cause of years lost due to ill health, disability or early death for this age group [3], (iii) it is hard for young people with SMI to recover function, resulting in impaired quality-of-life, medical morbidity and suicide, at a cost, for example in Canada, of over $50 billion per year.

Many gaps exist in our understanding of youth origins of SMI. First, we do not know how to identify youth at risk of SMI. Second, we do not know how best to define the stage of illness so that relevant, targeted preventive services can be offered. Third, we do not have the capacity to predict who will develop an SMI, what that illness might be, and what can be done to change its trajectory. Finally, we do not know how risk factors precipitate the neurobiological changes that determine who will develop a SMI. These are critically important questions regarding how, when and why youth transition from being healthy children to young adults with life-long illnesses.

We are studying a cohort of youth at various stages of risk for developing SMI by using a clinical staging model of mental disorders [4, 5]. The natural history of major mental disorders, such as psychosis, bipolar disorder and recurrent depression, is theorized to consist of transitions from being asymptomatic (i.e., having genetic risk but no symptoms) (stage 0) to a stage of undifferentiated general symptoms (stage 1a) to a worsening of existing symptoms or acquisition of new symptoms, whereby the person appears to have an attenuated form of a distinguishable mental disorder (stage 1b) until eventually (for some) a threshold diagnosis is reached (stages 2–4) (See Table 1). In other medical specialities, in particular oncology, [6, 7] staging models have been successful in integrating imaging, histology and molecular biology. Clinical staging can inform prognosis, clinical course and treatment, and assist with individualization of care but has not yet been tested in the field of mental health [4, 5]. Thus, our project is unique in that we will define our sample according to this stage model which allows for the inclusion of youth with transient mental illness who have important immediate health care needs and accompanying disability.

**Table 1:** Clinical Staging Framework for Mental Health Disorders [4]

Stage	Definition
0	No clinical symptoms • Increased risk of disorder (family history)
1a	Distress Disorder • No attenuated psychotic symptoms • Non-specific symptoms of anxiety or depression • Mild to moderate severity of symptoms • May include subjective/objective evidence of mild cognitive deficits. • Evidence of only recent or mild impacts of illness on social, educational or occupational function
1b	Attenuated Syndromes • Distress disorder plus at least one moderate-severe attenuated psychotic symptom (unusual thoughts, suspiciousness, perceptual abnormalities, grandiosity, disorganization) • Specific symptoms of anxiety or depression, brief hypomania or brief psychotic phenomena • May include subjective/objective evidence of at least moderate cognitive change • Moderate to severe impact of illness on social, education or employment functioning
2	Discrete Disorders • Discrete episodes of psychosis, mania or severe depression • Full threshold disorder with moderate-severe symptoms and persistence over time • Illness is having a major impact on social, educational or occupational functioning
3a-3c 4	Stages not Relevant for this Project Incomplete remission to multiple relapses Unremitting course of illness

This paper presents the methodology of our Brain Canada-funded project, the Adolescent Mental Health: Canadian Psychiatric Risk and Outcome (PROCAN) study in conjunction with  the Canadian Biomarker Integration Network in Depression (CAN-BIND) through the Ontario Brain Institute (OBI) that began in January 2015 with data collection beginning in April 2015. Our work addresses two key concerns in the field of youth mental health. First, the clinical presentation of youth with mental illness is frequently characterized by heterogeneous symptom patterns that are often comorbid with substance misuse. Early manifestations of potential illness are often brief or undifferentiated, and symptoms may emerge years before distinct diagnosable disorders [8]. These manifestations can be disabling even before they become full-blown disorders [9]. Because of a current emphasis on formal diagnosis at the individual and service level, youth with early symptoms are marginalized with delayed and/or limited treatment access. Even when young people receive “a diagnosis”, that diagnosis frequently changes [10–12], emphasizing the importance of studying the evolution of SMI broadly rather than in a diagnosis-specific manner.

The second key concern is that SMI has a multi-factorial aetiology, resulting from interactions among clinical, psychosocial and biological factors. In recent years, epidemiological studies, e.g. [13, 14], have demonstrated that the accumulation of stressors (trauma, abuse, bullying, etc.) and the early use of cannabis in adolescence are associated with an increased propensity for the development of SMI (in particular schizophrenia and mood disorders) [15]. These risk factors interact and may synergistically combine with pre-existing liabilities, including genetic and other biological factors.

### Study objectives

There are four main sets of objectives of the PROCAN project: clinical, neuroimaging, developing prediction models and defining peripheral biomarkers. The clinical objectives include improving our ability to identify youth at risk of SMI by determining clinical, social and cognitive factors associated with different stages of risk. Secondly, we aim to better understand factors that predict key outcomes, such as advancing disability, secondary substance misuse, non-participation in education and employment, and new self-harm. The imaging objectives first aim to identify structural and functional correlates of a predisposition to develop a SMI, and secondly, to understand how progression through the clinical stages of illness is associated with progressive brain changes. For our third set of objectives we aim first to develop a prediction model that predicts transition to illness or even the transition to a more serious stage of risk. We also aim to determine whether incorporating imaging data with clinical data improves the predictive value of the prediction model. Finally, we aim to explore potential peripheral biomarkers. This is being carried out in collaboration with CAN-BIND [16]. This collaboration involves collecting blood samples, according to the CAN-BIND protocol, for proteomic, epigenetic and genomic analyses, maximizing the opportunity to identify novel peripheral biomarkers of a predisposition to develop SMI.

From this cohort, clinical, social and cognitive data, as well as imaging data is gathered to create a multi-layered, comprehensive “snapshot” of these youth that will be employed to develop algorithms that predict the development of SMI.

## Methods

### Sample

Recruitment procedures include distributing materials describing the study and presentations to all clinical agencies that might be seeing youth who are help seeking, school counsellors and school social workers. We advertised widely in local papers and offered presentations to the general public and relevant youth groups. The sample is a cohort of 240 adolescents and young adults (aged 12–25, male and female) that includes youth with early mood symptoms or sub-threshold psychotic symptoms (symptomatic group; *n* = 160), youth at risk due to a family history of a SMI (family high risk (FHR); *n* = 40), and healthy controls (HC; n = 40). The stage of risk is defined based on the clinical staging model presented in Table 1.

Exclusion criteria for all participants include the following: (i) meet criteria for current or lifetime Axis I bipolar or psychotic disorder (other Axis I disorders will not be exclusionary as they may be precursors to mood or psychotic disorders); (ii) IQ < 70; (iii) past or current history of a significant central nervous system disorder or serious medical disorder; and (iv) current pharmacological treatment that would be considered as an adequate trial of treatment for a SMI.

For the symptomatic group (*N* = 160), we are recruiting approximately 80 individuals who meet criteria for stage 1a and 80 for stage 1b (see Table 1). Stage 1a will comprise those who meet criteria for distress disorder based on published ranges of the Kessler 10 (K-10) Distress Scale [17], a broad measure of psychological distress, with consideration of additional measures, e.g., Beck Depression Scale, Social Interaction Anxiety Scale (SIAS), Social Anxiety Scale (SAS) (see Table 2), to delineate anxiety and depression symptoms. Within this group, participants will have ratings on other measures to define their particular symptoms. For stage 1b participants, scores above threshold on measures such as the Scale of Prodromal Symptoms (SOPS), and the Calgary Depression Scale for Schizophrenia (CDSS), which identify those with clinical high-risk features for specific mental disorders, will be added to the K-10 score. In the FHR and HC groups, none of the participants will meet any of the criteria for stages 1a and 1b. The FHR group will include those with a first-degree relative with a psychotic disorder, bipolar disorder or recurrent mood disorder (stage 0). This distribution allows for a more heterogeneous sample, which not only fits better with our aims but also with recent research policy, in particular with the Research Domain Criteria [18] by addressing particular groups of “patients” that are not based on standard diagnoses. This “allows for a sample that provides appropriate variance along a dimension of interest” (p.813) [19].

**Table 2:** Schedule of Events

Assessment	Baseline	6 month	12 month	Transition
Demographics	**X**			
Family Interview for Genetic Studies	**X**			
Childhood Trauma Questionnaire [47]	**X**			
Family & Social Environment [48]	**X**			
Premorbid Adjustment Scale [49]	**X**			
Temperament [50]	**X**			
Wechsler Abbreviated Scale of Intelligence (WASI)	**X**			
MATRICS Cognitive Battery [51]	**X**		**X**	**X**
Scale of Prodromal Symptoms (SOPS) [52]	**X**	**X**	**X**	**X**
SCID	**X**	**X**	**X**	**X**
Calgary Depression Scale for Schizophrenia (CDSS) [53]	**X**	**X**	**X**	**X**
Beck Depression Scale [54]	**X**	**X**	**X**	**X**
Columbia Suicide Scale, [55]	**X**	**X**	**X**	**X**
Young Mania Scale [56]	**X**	**X**	**X**	**X**
Alcohol/Drug Use Scale [57]	**X**	**X**	**X**	**X**
Cannabis Use Measure [58]	**X**	**X**	**X**	**X**
Global Functioning (Social & Role) [59]	**X**	**X**	**X**	**X**
Social Interaction Anxiety Scale(SIAS); Social Anxiety Scale (SAS) [60]	**X**	**X**	**X**	**X**
Distress Scale [17]	**X**	**X**	**X**	**X**
Brief Core Schema Scales [61]	**X**	**X**	**X**	**X**
Life Events [62]	**X**	**X**	**X**	**X**
Ruminative Responses Subscale [63]	**X**	**X**	**X**	**X**
Snaith Hamilton Pleasure Scale [64]	**X**	**X**	**X**	**X**
Treatment Logs	**X**	**X**	**X**	**X**
BMI and blood pressure	**X**	**X**	**X**	**X**
Neuroimaging	**X**		**X**	**X**
Biomarkers - Blood draws for DNA, RNA and plasma protein analyses	**X**		**X**	**X**

There is no standard formula for calculating the sample size needed to develop a prediction model. Therefore, we followed the guidelines from Peduzzi et al. [20]To maintain accuracy and precision and to minimize bias, we plan to use the number of events per variable of 10 as the guiding rule. In this study 200 participants with sub-threshold symptoms and/or family history will be recruited. Assuming that the risk of transition from the clinical high-risk stage to SMI is 35% over 2.5 years, [21, 22] 70 events would be expected, which ensure sufficient power for developing a model with 6 predictors.

Two hundred participants are being recruited from the University of Calgary site, with an additional 40 being recruited from Sunnybrook Health Sciences Centre, in Toronto.

The study was approved by The University of Calgary Conjoint Health Research Ethics Board (ID:REB 14–1710) and the Sunnybrook Research Ethics Board (ID:100–2015). Participants provided informed consent or assent (parental informed consent for minors).

### Design

Individuals who respond to recruitment efforts are screened by telephone and, if suitable, invited to an in-person assessment to evaluate inclusion and exclusion criteria. Those who are eligible and interested sign informed consent; in the case of minors, this is obtained from parents/guardians. Standard procedures are followed to ensure confidentiality. Participants are assessed at baseline on all measures, followed by short clinical assessments at 6 and 12 months. Scanning and blood draws occur in conjunction with baseline and 12-month assessments. If, at any point, a participant makes a transition to a SMI, we repeat the imaging and blood draws in addition to the clinical assessment.

### Clinical assessments

Clinical measures were selected based on their relevance, excellent psychometric properties, utility as repeated measures, suitability for adolescents, and participant tolerability. The Structured Clinical Interview for DSM-V Disorders (SCID) [23] is used to determine the presence of any Axis I disorder. All assessment measures are presented in the Schedule of Events (Table 2) and include the following domains: psychopathology; social and role functioning; past and current stressors; substance use; cognition; and beliefs/attitudes. Comprehensive treatment logs of all past and current psychosocial and pharmacological treatments are recorded. We also measure Body Mass Index (BMI) and blood pressure in order to better understanding associations between SMI and medical comorbidity.

All clinical raters, under the supervision of Drs. Addington and Goldstein, are required to complete a rigorous protocol for training, standardization of procedures and maintenance of reliability developed for previous multi-site projects [24]. At weekly conference calls with all raters, led by Dr. Addington, written comprehensive vignettes will be presented for each participant to determine consensus on that participant’s clinical stage.

### Neuroimaging

The neuroimaging component aims to determine whether baseline brain structure and function can distinguish youth who will develop SMI from those who will not. Algorithms exist that identify individual patients with neurologic and psychiatric disorders based on integrated neuroimaging and clinical data. This approach involves identifying a set of features from the data collected that show significant differences between symptomatic participants and controls. Such features may be drawn from clinical data (e.g., standardized scales), structural MRI or diffusion MRI (e.g., regional grey matter volume, white matter integrity), functional data (e.g., resting-state connectivity among brain regions of interest or task-related signal changes) or some combination of all three. These feature sets are then used to train automated pattern-recognition algorithms such as Support Vector Machines (SVMs), enabling them to classify individual scans as normal or pathological. Such methods have been successfully applied to distinguish healthy controls from patients with schizophrenia, bipolar disorder, Alzheimer’s disease, and major depressive disorder [25–27]. The same techniques are expected to prove useful in predicting outcome in patients at risk for developing SMI [28].

Functional Magnetic Resonance Imaging (fMRI) will be collected both while participants are resting passively (resting state; rs-fMRI) and during a series of tasks. The fMRI tasks have been chosen to engage cognitive, cognitive-emotional networks and cortico-striatal reward circuits. Analyses will assess task-dependent blood oxygenation level-dependent (BOLD) signal change and functional connectivity in these regions. fMRI will assess changes in functional connectivity in striatal-hippocampal, amygdala-PFC and cortico-thalamo-striatal networks.

Diffusion Tensor Imaging (DTI) and advanced diffusion models (e.g. Neurite orientation dispersion and density imaging (NODDI) and diffusion kurtosis imaging) will be used to examine changes in structural links between these and other regions. In psychiatric illness, wide-spread reductions in white matter (WM) integrity have been observed; however, the stage at which these abnormalities first appear, and whether they are correlates of illness progression, as opposed to an increased vulnerability remains unclear. In individuals at risk for SMI, for instance those at risk for psychosis, widespread WM aberrations have been observed in multiple brain regions [29], with fronto-temporal and fronto-limbic connections, including the superior longitudinal and uncinate fasciculus and corpus callosum particularly implicated [30].

Arterial spin labeling (ASL) perfusion MRI measures regional cerebral blood flow (rCBF), and may be used to study subtle brain perfusion changes occurring in psychiatric illnesses. [31] Abnormalities in microvasculature can result in functional deficits because of the coupling between neuronal firing rates and blood oxygen consumption [32], thus providing useful knowledge of brain areas with disease-related abnormalities in cerebral microvasculature function. Perfusion patterns may hold promise as objective biomarkers by assessing whether rCBF patterns differ in at-risk groups. Adolescents with mood disorders, for example, appear to differ from healthy adolescents on rCBF in executive, affective and motor networks [33].

#### MRI protocol

Participants undergo 3 T MRI at baseline and the 12-month follow-up. Sessions consist of structural and functional neuroimaging sequences comprising the following:

1. Whole-brain T1-weighted anatomical scan at 1mm^3^ resolution;
2. Whole-brain diffusion imaging using 45 gradient directions and 32 gradient directions at UCA and SB, respectively, each at 2 b-values (2500 and 1000 s/mm^2^) with 16 images at b = 0 s/mm^2^ for tensor construction;
3. 3D pseudo continuous ASL perfusion MRI sequence;
4. A 10-min resting-state functional neuroimaging scan during which participants are instructed to keep their eyes open and focus on a fixation cross. Images are obtained using a whole-brain T2*-sensitive BOLD echo planar imaging (EPI) series;
5. Task-based functional neuroimaging (BOLD series) which focus on the integration of emotional and cognitive function (emotional go/no go task), and categorical learning task (monetary incentive delay task) along with a working memory task.

See Additional file 1: Material 1 for details of the neuroimaging tasks and Additional file 2: Material 2 for details of neuroimaging parameters for both image acquisition sites and scanners.

### Peripheral biomarkers

This study affords the opportunity to collect biochemical information from carefully characterized clinical subjects that can cast light on the neural mechanisms by which exposure to stress and substance use during adolescence promote the development of SMI. We collect DNA, RNA and plasma protein samples at specific intervals (Table 2) and transfer these to CAN-BIND for storage. Under the rubric of CAN-BIND we will complete genomic, epigenetic and proteomic analyses. These analyses may include profiling of mRNA and miRNA, histone modifications, methylation status across the genome wide assessment of DNA. Other possible analyses are oxidative damage to DNA, [34] and Selected Reaction Monitoring proteomic assays that can allow systematic exploration of biological pathways thought to be involved in SMI.

### Prediction models

We will first develop and validate a prediction algorithm that includes demographic, clinical and psychosocial predictors. Transition to SMI over the study period will be the outcome variable. Such an algorithm can be feasibly employed at the clinical assessment stage. Next, we will develop a second algorithm that includes neuroimaging data along with demographic, clinical and psychosocial predictors. We hypothesize that this algorithm will have greater discriminative power than the first one, while acknowledging that this has not been the case in all reports. The rationale for having two separate prediction models is that brain imaging is expensive and may not be an efficient use of resources to conduct such assessments in every patient. If individuals are found to be at high risk based on the first algorithm, neuroimaging may be recommended. Using the second model, the risk of developing SMI can be more precisely and objectively predicted.

#### General strategies

The longitudinal data may (i) have missing values, (ii) have highly correlated predictors, and (iii) violate the assumptions of a particular modelling approach. For variables with missing values, the mechanisms of missing data will be examined to determine whether a multiple imputation method is appropriate. For highly correlated predictors, we will keep the one that is clinically relevant, feasible for routine data collection, and that adds more predictive power to the model. For model development, we will use the Cox proportional hazard model, which is a time-to-event model that has been widely used in prediction research. If the assumption of proportionality is violated, we will use other approaches, including the accelerated failure time model, which does not require the assumption of proportionality [35], the repeated measures discriminant analysis model, which can accommodate within-predictor and between-predictor correlations, or a machine learning approach that can compare performance of models developed using different approaches.

#### First prediction algorithm

Our prediction model will include six predictors or less so that it can be of practical use and minimize potential problems related to over-fitting and instability. The potential predictors will be the summary scores from the selected instruments that measure clinical symptoms, social functioning, substance use, cognition and adjustment, as outlined in Table 2. The initial selection of candidate predictors will be informed by the statistical analyses of the cohort project, literature review and knowledge about the clinical relevance of the variables. We will use combined procedures of forward and backward selection for model development, as each individual approach has its own limitations [36]. We will first include current clinical stage and cannabis use in the model. We will then examine other candidate predictors to determine whether they improve the model’s discriminative power and calibration with data by comparing the difference between the C statistics of the models with and without the variable. Calibration measures how closely predicted outcomes agree with actual outcomes. For this, we will use the Hosmer-Lemeshow test to compare the differences between mean predicted and actual event rates. We will also use the method of Net Reclassification Improvement [37] to examine whether adding a particular variable could correctly reclassify participants into appropriate categories. Once the prediction model is developed, we will conduct bootstrap validation by repeatedly resampling from the original data with replacement and estimate the accuracy of the prediction algorithm. Because similar demographic, clinical and psychosocial data have been collected in the North American Prodrome Longitudinal Study (NAPLS) study, we will use the data from the US sites of the NAPLS consortium to validate the first prediction algorithm. The proposed analytic procedures are consistent with the framework of Prognosis Research Strategy [38, 39] and methodology that is commonly used in prediction research [37, 40].

#### Second prediction algorithm

This algorithm will be used for more precise and objective prediction with imaging markers in those at high-risk based on the first model. The neuroimaging group will identify a set of structural and functional features that differentiate healthy controls, converters and non-converters. We will use the same model-fitting strategies as described above. We will examine whether adding the predictors to the first model will improve discrimination without compromising calibration. If the second model can yield more precise prediction than the first one, we will conduct a preliminary analysis on the cost-benefit of using the two models for prediction and intervention planning.

Finally, if potentially relevant neurochemical biomarkers are later identified these will be added to the second prediction algorithm as described above.

## Discussion

This paper has described the details and methods of the Adolescent Mental Health: Canadian Psychiatric Risk and Outcome (PROCAN) study. This project has several unique features: (i) using a staging model to define level of risk in a diagnostically unconstrained sample; (ii) developing prediction algorithms for transition to illness that could have clinical utility; and (iii) incorporating neuroimaging data into clinical algorithms to assess the additional predictive value of neuroimaging.

Prediction algorithms are tools that combine a key set of known predictors from which the risk of future disease can be calculated for individual patients [39]. They aid health professionals and individuals in making informed decisions. Well-known examples include the Framingham risk prediction algorithms for cardiovascular disease [41] and for cancer risk. [42, 43] However, their use for psychiatric care lags far behind cardiology and oncology [44–46]. Moreover, no attempts have been made to investigate the roles of neurobiological and genetic markers in prediction models for SMI.

There are several important outcomes that may result from this project. First results may lead to an improved clinical staging model for SMI and prediction algorithms that can be used by health care providers as decision-support tools in their practices. Secondly, our research will lead to a greater understanding of clinical, social and cognitive factors associated with the clinical stages of development of a SMI, as well as new insights from neuroimaging and later neurochemical biomarker studies regarding predisposition to SMI development and progression through the clinical stages of illness. Our results will also be used to inform health policies, health education and promotion activities that are related to the predictors in the algorithms. Serious mental illness results in impaired quality-of-life, medical morbidity and suicide. Improved identification of youth at risk represents our best chance at providing effective, appropriate and cost-effective treatment to each young person who needs help.

## Additional files

Additional file 1: Material 1 neuroimaging tasks.pdf. Details of the neuroimaging parameters for both image acquisition sites and scanners are in file (PDF 191 kb)

Additional file 2: Material 2 neuroimaging parameters.pdf. (PDF 183 kb)

## Abbreviations

ASL: Arterial spin labeling
BMI: Body mass index
BOLD: Blood oxygenation level-dependent
CAN-BIND: Canadian biomarker integration network in depression
CDSS: Calgary depression scale for schizophrenia
DTI: Diffusion tensor imaging
EPI: Echo planar imaging
FHR: Family high risk
fMRI: Functional magnetic resonance imaging
HC: Healthy controls
K-10: Kessler 10 distress scale
NAPLS: North American prodrome longitudinal study
NODDI: Neurite orientation dispersion and density imaging
OBI: Ontario brain Institute
PROCAN: Adolescent mental health: Canadian psychiatric risk and outcome
rCBF: Regional cerebral blood flow
rs-fMRI: Resting state functional magnetic resonance imaging
SAS: Social anxiety scale
SCID: Structured clinical interview for DSM-V disorders
SIAS: Social interaction anxiety scale
SMI: Serious mental illness
SOPS: Scale of prodromal symptoms
SVMs: Support vector machines
WM: White matter
