# Supplementary material for: Youth at-risk for serious mental illness: methods of the PROCAN study
Source: BMC Psychiatry. 2018 Jul 5;18:219. doi: 10.1186/s12888-018-1801-0 (PMC6034268; doi:10.1186/s12888-018-1801-0)
Supplement: Supplementary file 1 — Material 1 neuroimaging tasks.pdf. Details of the neuroimaging parameters for both image acquisition sites and scanners are in file (PDF 191 kb) [file 12888_2018_1801_MOESM1_ESM.pdf]

Additional Material 1: Neuroimaging Tasks

Affective Go-NoGo Response Inhibition task

We employed a modified version of an affective Go/NoGo paradigm. Go/NoGo paradigms involve frequently presented “go” cues to which participants respond as fast as possible, and infrequently presented “no-go” cues to which participants are instructed to not respond. The frequency of “go” cues (typically ≥70%) creates a pre-potent tendency to respond, which must then be inhibited for “no-go” cues, thereby providing a measure of response inhibition (Schulz et al., 2007). The emotional Go/NoGo task additionally allows for the analysis of performance in response to cues of different emotional valences (e.g., happy versus angry) thereby providing not only a measure of behavioral inhibition, but also of the emotional modulation of this inhibition. Assessing the neural signature of an affective Go-NoGo task has revealed activation associated with correct no-go trials in the temporo-parietal junction as well as inferior-frontal and superior-parietal regions. Assessment of the emotional valence of trials, e.g., the comparison of emotional versus neutral faces revealed valence-dependent activation in the amygdala, anterior insula and posterior mid-cingulate cortex (Schulz et al., 2009).
During this modified affective Go/NoGo task(Schulz et al., 2009). During this modified Go-NoGo participants are presented with a square symbol or a circle symbol. They are instructed to press a response button every time a circle appears, and withhold their button press when a square appears. If participants make a mistake (i.e., pressing a button for the square), the square turns red to remind them not to respond. When the participant correctly presses for a circle, the circle changes from black to gray. In the background there are neutral (mildly positive) and angry faces. Participants are not expected to respond to the faces; thus, emotion processing is implicit. The onset of the face precedes the square or circle, and this onset time is jittered.

Monetary Incentive Delay (MID) task/Reversal Learning Task

In MID-type tasks trials feature cues signaling potential monetary rewards, losses or no incentive, a delay anticipation period, a target stimulus (to which participants respond with a speeded button press), and an outcome period during which rewards or penalties are delivered. During these tasks, anticipation of reward is associated with activation of the ventral striatum, including the nucleus accumbens (NAcc); receipt of reward is associated with patterns of activation in medial and ventromedial prefrontal cortical regions (B Knutson, Fong, Adams, Varner, & Hommer, 2001; Brian Knutson, Fong, Bennett, Adams, & Hommer, 2003). Anticipating rewards, or losses, evokes increased arousal and attention, as well as various emotional states. This is expected to elicit activity in brain regions associated with cognitive control and emotion–attention interactions, e.g., the anterior cingulate cortex (ACC; (Bush et al., 2002); but may also elicit activation in brain regions related to processing the hedonic value of such stimuli, e.g., medial-orbito-frontal cortex (mOFC) (Kim, Shimojo, & O’Doherty, 2006). In addition, hippocampal and striatal learning systems[1;2] interact during goal-directed behaviour and will be assessed in parallel by modulating the timing of feedback during a categorical learning task: delayed feedback favours hippocampus-based learning, while rapid feedback

favours dopaminergic/striatal learning [ADD citation: http://shohamylab.psych.columbia.edu/content/papers/Jneuro_Foerde_Shohamy.pdf]. During this task, participants must try and press the response button during the time that a red square appears on the screen - the duration of the square is very brief - and trials are adapted so that participants are successful for about 50% of the time. If participants are successful in pressing the button during the presentation of red square it is scored as a HIT - if they respond too late - it is scored a MISS. Prior to each trial, participants are informed whether a HIT would result in a dollar gain (1$), or in no payout (0$ gain). Participants must respond as quickly as possible, however, if they respond too quickly (i.e., trying to anticipate the square) they will also score a MISS and will be informed that they have responded too early.

Working memory task

The Working Memory task, specifically an N-back task, was chosen from the Human Connectome Project (http://www.humanconnectomeproject.org), because this task has been associated with reliable brain activations across participants (Drobyshevsky, Baumann, & Schneider, 2006) and across time (Caceres, Hall, Zelaya, Williams, & Mehta, 2009). In this task, blocks of trials are presented showing pictures of faces, places, tools and body parts. These stimuli were chosen because they reliably engage distinct cortical regions (Downing, Jiang, Shuman, & Kanwisher, 2001; Peelen & Downing, 2005). Half of the blocks use a 2-back working memory task (respond 'target' whenever the current stimulus is the same as the one two places back), the other half use a 0-back working memory task (a target cue is presented at the start of each block, and the person must respond 'target' to any presentation of that stimulus during the block). A 2.5s cue indicates the task type (and target for 0-back) at the start of the block. On each trial, the stimulus is presented for 2s, followed by a 500ms inter-trial interval (ITI). Each block contains 10 trials, of which 2 are targets, and 2–3 are non-target lures (e.g., repeated items in the wrong n-back position, either 1-back or 3-back). The inclusion of lures is critical to ensure that the participants are using an active memory approach to the task and allows one to assess conflict related activity as well as error related activity (Barch et al., 2013).

References

Barch, D. M., Burgess, G. C., Harms, M. P., Petersen, S. E., Schlaggar, B. L., Corbetta, M., … WU-Minn HCP Consortium. (2013). Function in the human connectome: task-fMRI and individual differences in behavior. *NeuroImage*, *80*, 169–89. https://doi.org/10.1016/j.neuroimage.2013.05.033

Bush, G., Vogt, B. A., Holmes, J., Dale, A. M., Greve, D., Jenike, M. A., & Rosen, B. R. (2002). Dorsal anterior cingulate cortex: a role in reward-based decision making. *Proceedings of the National Academy of Sciences of the United States of America*, *99*(1), 523–8. https://doi.org/10.1073/pnas.012470999

Caceres, A., Hall, D. L., Zelaya, F. O., Williams, S. C. R., & Mehta, M. A. (2009). Measuring fMRI reliability with the intra-class correlation coefficient. *NeuroImage*, *45*(3), 758–68. https://doi.org/10.1016/j.neuroimage.2008.12.035

Downing, P. E., Jiang, Y., Shuman, M., & Kanwisher, N. (2001). A cortical area selective for visual processing of the human body. *Science (New York, N.Y.)*, *293*(5539), 2470–3. https://doi.org/10.1126/science.1063414

Drobyshevsky, A., Baumann, S. B., & Schneider, W. (2006). A rapid fMRI task battery for mapping of visual, motor, cognitive, and emotional function. *NeuroImage*, *31*(2), 732–44. https://doi.org/10.1016/j.neuroimage.2005.12.016

Ho, T. C., Wu, J., Shin, D. D., Liu, T. T., Tapert, S. F., Yang, G., … Yang, T. T. (2013). Altered cerebral perfusion in executive, affective, and motor networks during adolescent depression. *Journal of the American Academy of Child and Adolescent Psychiatry*, *52*(10), 1076–1091.e2. https://doi.org/10.1016/j.jaac.2013.07.008

Kim, H., Shimojo, S., & O'Doherty, J. P. (2006). Is avoiding an aversive outcome rewarding? Neural substrates of avoidance learning in the human brain. *PLoS Biology*, *4*(8), e233. https://doi.org/10.1371/journal.pbio.0040233

Knutson, B., Fong, G. W., Adams, C. M., Varner, J. L., & Hommer, D. (2001). Dissociation of reward anticipation and outcome with event-related fMRI. *Neuroreport*, *12*(17), 3683–7. Retrieved from http://www.ncbi.nlm.nih.gov/pubmed/11726774

Knutson, B., Fong, G. W., Bennett, S. M., Adams, C. M., & Hommer, D. (2003). A region of mesial prefrontal cortex tracks monetarily rewarding outcomes: characterization with rapid event-related fMRI. *NeuroImage*, *18*(2), 263–72. Retrieved from http://www.ncbi.nlm.nih.gov/pubmed/12595181

Kuschinsky, W. (1991). Coupling of function, metabolism, and blood flow in the brain. *Neurosurgical Review*, *14*(3), 163–8. Retrieved from http://www.ncbi.nlm.nih.gov/pubmed/1944930

Peelen, M. V, & Downing, P. E. (2005). Within-subject reproducibility of category-specific visual activation with functional MRI. *Human Brain Mapping*, *25*(4), 402–8. https://doi.org/10.1002/hbm.20116

Schulz, K. P., Clerkin, S. M., Halperin, J. M., Newcorn, J. H., Tang, C. Y., & Fan, J. (2009). Dissociable neural effects of stimulus valence and preceding context during the inhibition of responses to emotional faces. *Human Brain Mapping*, *30*(9), 2821–2833. https://doi.org/10.1002/hbm.20706

Schulz, K. P., Fan, J., Magidina, O., Marks, D. J., Hahn, B., & Halperin, J. M. (2007). Does the emotional go/no-go task really measure behavioral inhibition? Convergence with measures on a non-emotional analog. *Archives of Clinical Neuropsychology : The Official Journal of the National Academy of Neuropsychologists*, *22*(2), 151–60. https://doi.org/10.1016/j.acn.2006.12.001

Théberge, J. (2008). Perfusion Magnetic Resonance Imaging in Psychiatry. *Topics in Magnetic Resonance Imaging*, *19*(2), 111–130. https://doi.org/10.1097/RMR.0b013e3181808140
