# Supplementary material for: Youth at-risk for serious mental illness: methods of the PROCAN study
Source: BMC Psychiatry. 2018 Jul 5;18:219. doi: 10.1186/s12888-018-1801-0 (PMC6034268; doi:10.1186/s12888-018-1801-0)
Supplement: Supplementary file 2 — Material 2 neuroimaging parameters.pdf. (PDF 183 kb) [file 12888_2018_1801_MOESM2_ESM.pdf]

	SunnyBrook	UCalgary
	Phillips 3.0T Achieva	GE 3.0 Tesla Discovery MR750
**T1w Sag Struct**		
TR (ms)	6.5	6.4
TE (ms)	3	2.8
Flip Angle	8	15
Pixel Bandwidth	241	260
Matrix Size	240 x 240	240 x 240
Slice Thickness (mm)	1	1
Voxel Size (mm)	1 x 1 x 1	1 x 1 x 1
Number of slices	155	180
**DTI 31 dirs 6T2 b1000**		
TR (ms)	9000	8000
TE (ms)	94.00	93.60
Flip Angle	90.00	90.00
Pixel Bandwidth	3256.00	1953.12
Matrix Size	96.00	100.00
Voxel Size (mm)	2.2 x 2.2 x 2.2	2.2 x 2.2 x 2.2
Number of slices	59.00	58.00
Number of Diffusion Directions	32.00	45.00
Diffusion b value (s/mm^2)	1000 and 2500	1000 and 2500
Diffusion b=0 images	25.00	16
**PD-T2w Ax Struct**		
TR (ms)	5500-6500	5428
TE (ms)	88	7.528; 90.336
Flip Angle	125.00	125.00
Pixel Bandwidth	188.00	325.52
Matrix Size	240 x 190	192 x 192
Voxel Size (mm)	1.25 x 1.37 x 2.5	1.25 x 1.25 x 1.25
Number of slices	58	58
**Arterial Spin Labeling**		
TR (ms)	Not run at SunnyBrook due to time constraints	5046.00
TE (ms)		11.09
Flip Angle		111.00
Inversion time (ms)		2025
Pixel Bandwidth		976.56
Matrix Size		512 x 8
Slice Thickness (mm)		3.00
MR acquisition type		3D
Reconstruction Diameter		192.00

fMRI - Resting State		
TR (ms)	2000	2000
TE (ms)	30	30
FOV	256	256
Image Dimension	64 x 64 x 36	64 x 64 x 36
Flip Angle	75	75
Pixel Bandwidth	3589	7812
Matrix Size	64 x 64	64 x 64
Voxel Size (mm)	4 x 4 x 4	4 x 4 x 4
Number of slices	40.00	36.00
Time/Duration of scan (minutes)	10:00	10:00
**fMRI - Go-NoGo**		
TR (ms)	2000	2000
TE	30	30
FOV	256	256
Image Dimension	64 x 64 x 36	64 x 64 x 36
Flip Angle	75	75
Pixel Bandwidth	3589	7812
Matrix Size	64 x 64	64 x 64
Voxel Size (mm)	4 x 4 x 4	4 x 4 x 4
Number of slices	36.	36
Time/Duration of scan (minutes)	10:00	10:00
**fMRI - Anhedonia**		
TR (ms)	2000	2000
TE	30	30
FOV	256	256
Image Dimension	64 x 64 x 36	64 x 64 x 36
Flip Angle	75	75
Pixel Bandwidth	3589	7812
Matrix Size	64 x 64	64 x 64
Voxel Size (mm)	4 x 4 x 4	4 x 4 x 4
Number of slices	36	36
Time/Duration of scan (minutes)	11:40	11:40
**fMRI - Working Memory**		
TR (ms)	2000	2000
TE (ms)	30	30
FOV	256	256
Image Dimension	64 x 64 x 36	64 x 64 x 36
Flip Angle	75	75
Pixel Bandwidth	3589	7812

Matrix Size	64 x 64	64 x 64
Voxel Size	4 x 4 x 4	4 x 4 x 4
Number of slices?	36	36
Time/Duration of scan (minutes)	5:10	5:10
**Software version**	3.2.2,3.2.2.0	25,LX,MR Software release:DV25.0_R02_1549.b

Supplementary Material 2: Imaging sequences and parameters
